# An Ultrahigh Sensitivity Acetone Sensor Enhanced by Light Illumination

**DOI:** 10.3390/s18072318

**Published:** 2018-07-17

**Authors:** Heng Zhang, Hongwei Qin, Chengyong Gao, Jifan Hu

**Affiliations:** School of Physics, State Key Laboratory for Crystal Materials, Shandong University, Jinan 250100, China; 201411433@mail.sdu.edu.cn (H.Z.); gchy@sdu.edu.cn (C.G.)

**Keywords:** acetone vapor, gas sensor, Au:SmFe_0.9_Zn_0.1_O_3_, ultralow concentration

## Abstract

Au:SmFe_0.9_Zn_0.1_O_3_ is synthesized by a sol-gel method and annealed at 750 °C. Through XRD, SEM and XPS analysis methods, the microstructure of the material has been observed. The average particle size is about 50 nm. The sensor shows a high sensitivity toward acetone vapor. As the relative humidity increases, the resistance and sensitivity of the sensor decline. To obtain a low optimum operating temperature, light illumination with different wavelengths has been introduced. The sensitivity toward acetone is improved at lower operating temperature when the sensor is irradiated by light. The smaller the wavelengths, the better the sensitivity of the sensor. Compared with other gases, the sensor shows excellent selectivity to acetone vapor, with better sensitivity, selectivity and stability when under light illumination.

## 1. Introduction

Acetone is an important chemical material for industrial applications in many areas [1,2]. It is a colorless and transparent liquid with an aromatic odor. Its cheapness and practicality greatly increase the application value in the chemical industry field. However, acetone is easy flammable and volatile, and will explode when exposed to fire. Acetone has some poisonous effects and will cause some harm to our body. It can cause headache and nausea, and even harm peoples’ central nervous system when subjected to long-term exposure [3]. More than that, acetone exists at ultralow concentrations in exhaled breath. The concentrations of acetone in exhaled breath from healthy and diabetic patients are different [4], which can be used to judge whether a person is diabetic or not. Therefore, developing a sensor that can detect low or ultralow concentrations of acetone is essential and necessary [5,6,7,8,9,10].

Some detection methods are already used in hospitals or by special inspection agencies, such as quartz crystal microbalances and fiber-optic sensors. However, these analytical tools are inconvenient or expensive at any time or anywhere. People need gas sensors with high sensitivity, stability and low cost that can provide a chance to detect the concentration of acetone in exhaled breath or in the air around us. In the past decades, metal semiconductor oxides have attracted much attention for their high sensitivity and stability toward reducing gases. Examples such as TiO_2_ [11,12,13], NiO [14,15], ZnO [16,17,18,19], Co_3_O_4_ [20], Fe_2_O_3_ [21,22], WO_3_ [23,24,25], and SnO_2_ [26,27,28,29,30] are reported as acetone sensors with good performance. In recent years, the perovskite structure (ABO_3_) also showed good sensitivity, stability and selectivity toward acetone, with examples such as LaFeO_3_ [31,32] Yb_1−x_Ca_x_FeO_3_ [33] LaNi_1−x_Ti_x_O_3_ [34] SmFeO_3_ [35], SmFe_1−x_Mg_x_O_3_ [36], NdFeO_3_ [37] and La_0.75_Ba_0.25_FeO_3_ [38]. Noble metal doping in the perovskite structure (ABO_3_) toward reducing gases have been reported, and a higher sensitivity is common [39,40,41,42,43,44,45,46,47,48,49,50]. Inspired by this, Au:SmFe_0.9_Zn_0.1_O_3_ has been measured for high sensitivity toward acetone vapor.

At first, the response of SmFe_1−x_Zn_x_O_3_ has been measured, and SmFe_0.9_Zn_0.1_O_3_ shows better sensitivity, however, the SmFe_0.9_Zn_0.1_O_3_ sensor is unable to meet our requirements that a sensor display high sensitivity, good selectivity and stability, and can detect ultralow concentrations of acetone for more extensive application in many areas. Therefore, x% Au doping in the SmFe_0.9_Zn_0.1_O_3_ have been prepared, and the sensor when x = 2 shows a quite high sensitivity at 200 °C. Au:SmFe_0.9_Zn_0.1_O_3_ is measured toward 0.01–1 ppm acetone vapor in this work. As the relative humidity in air increases, the sensitivity and resistance decrease. For reducing the optimum operating temperature, light illumination with different wavelengths was introduced during the measurementd. Higher sensitivity and lower optimum operating temperature toward acetone vapor is shown when the sensor irradiated by light, and the smaller the wavelength, the better the sensitivity. The dynamic resistance curves have been researched and the mechanism whereby the sensitivity can be improved by light illumination has been approximately clarified. The stability of sensors is also measured every three days for a month. The sensor shows high sensitivity, good selectivity and stability compared to the other materials that have been reported for this use.

## 2. Materials and Methods

### 2.1. Preparation

Nanocrystalline powders of 2% Au doped SmFe_0.9_Zn_0.1_O_3_ were prepared by the sol-gel method. Firstly, a stoichiometric ratio of samarium oxide, ferric nitrate, zinc nitrate, PEG (molecular weight 20,000) and nitric acid (all analytically pure) was weighed and mixed in deionized water. Then, HNO_3_ was added to adjust the pH to 1.5~2. Subsequently, the solution was heated to 80 °C in a water bath with continuous stirring for two days to obtain a highly viscous solution; the solution became a gel a few hours later. The gel was dried in a baking box at 120 °C for 24 h to obtain dry, foamy powders before being ground to a fine powder. The powder was then annealed in an oven at 750 °C for 4 h after preheating at 400 °C for 2 h. Subsequently, a fraction of the powder was mixed with an appropriate amount of AuCl_2_ and ground for half an hour. Next, the mixture was annealed in an oven at 750 °C after which SmFe_1−x_Zn_x_O_3_ with x wt % Au doping was obtained.

### 2.2. Fabrication

**Figure 1:**
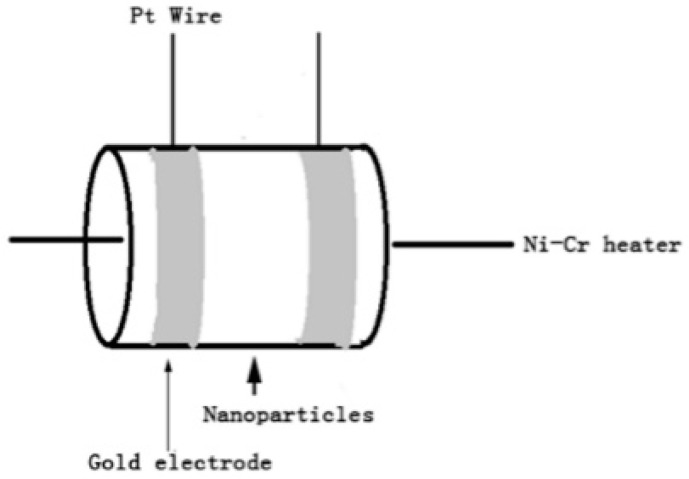
Schematic structure of the gas sensor.

The Au:SmFe_0.9_Zn_0.1_O_3_ was blended with deionized water to obtain a paste. The sensors were fabricated by coating the paste onto a ceramic tube with an approximately 2 mm external diameter, 8 mm length and 1.6 mm internal diameter with two electrodes. Figure 1 shows the schematic structure of the sensor. It can be seen that a pair of gold electrodes connected with Pt wire were installed at each end of the ceramic tube, between the two gold electrodes was the area which used for coating nanoparticles. A Ni-Cr heating wire used as a heat supply source was inserted into the ceramic tube to control the operation temperature of the gas sensor. The ready-made sensors were dried at 240 °C for 18 h to improve stability and repeatability. For gas test studies, a static gas chamber was used at atmospheric pressure and room temperature. A micro-injector was used to control the gas concentration after drawing in a suitable amount of liquid acetone. A characterization system (WS-30A, Wei Sheng Electronic Technology Co., Ltd., Zhengzhou, China) was used to measure the gas-sensing performance of the sensors. The entire experimental procedure was carried out at room temperature. The sensing response *S* (R_g_/R_a_) was used to define the response of the sensor. R_a_ is the resistance of the material in air and R_g_ is the resistance of the gas being tested. The response time was defined as the time taken to reach 90% of the maximum response, and the recovery time was defined as the time taken to reach 10% of the maximum response.

### 2.3. Gas-Sensing Test

A characterization system WS-30A (Wei Sheng Electronic Technology Co., Ltd., Zhengzhou, China) was used to measure the gas-sensing performance of the sensors. The characterization system (WS-30A) we used has been upgraded and modified according to our experimental needs by adding the irradiation function, filter function, etc. The static test-gas chamber was a 20 L covered glass container, and the temperature of the sensor was controlled by a computer. For the measuring concentration of acetone vapor, a calculated amount of liquid acetone using the ideal gas equation. During the entire measurement process, two fans were always turned on to volatile the acetone in the test-gas chamber. Cold LED lights with a power consumption of 50 mW were the light source. The distance from the LED to the sensor was approximately 15 cm. Light filters (10 cm length, 10 cm width and 0.3 cm thick) for different wavelengths were used for the LEDs and sensors to filter the light. The light from the LED was passed through a filter to obtain light with a special wavelength to irradiate the sensor.

### 2.4. Characterization

The structure of the final powder was characterized by X-ray diffraction (XRD) using CuKα radiation at room temperature. The Au element distribution across the semiconductor was analyzed by X-ray photoelectron spectroscopy (XPS). The microstructure of 2 wt % Au-doped SmFe_0.9_Zn_0.1_O_3_ was observed by field-emission scanning electron microscopy (FE-SEM).

## 3. Result and Discussion

**Figure 2:**
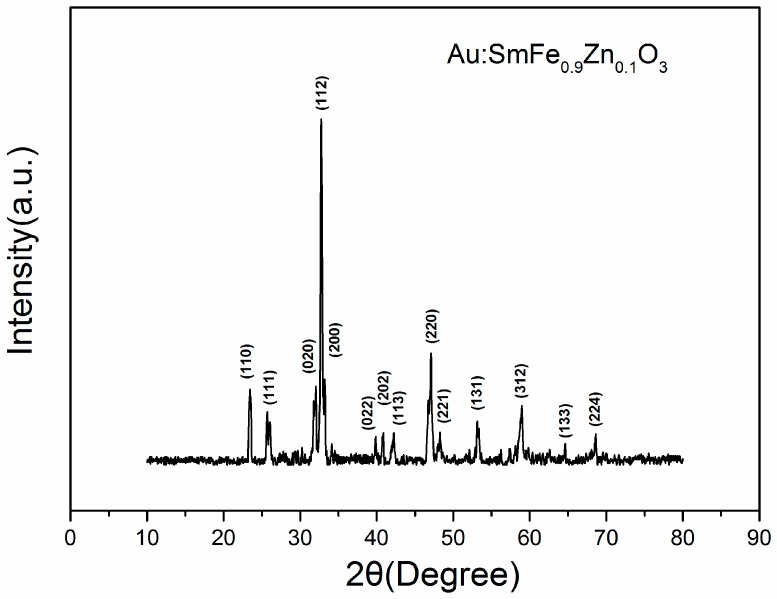
The XRD patterns of Au:SmFe_0.9_Zn_0.1_O_3_ annealed at 750 °C.

**Figure 3:**
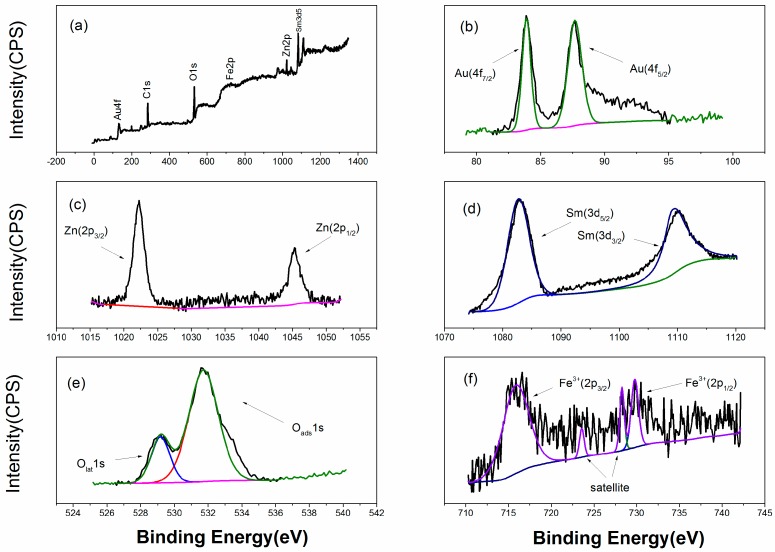
XPS spectra of Au:SmFe_0.9_Zn_0.1_O_3_. (**a**) XPS wide scans; (**b**) Au 4f; (**c**) Zn 2p; (**d**) Sm 3d; (**e**) O 1s; (**f**) Fe 2p.

**Figure 4:**
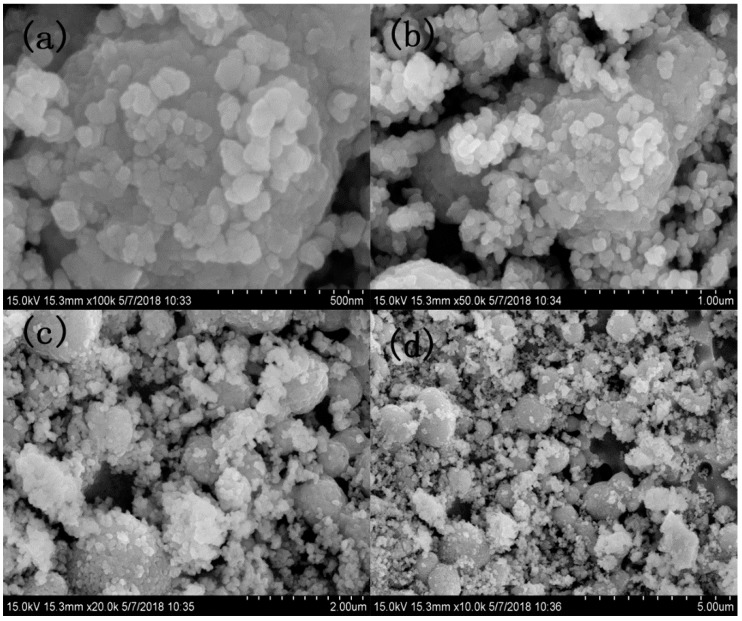
SEM micrographs of Au:SmFe_0.9_Zn_0.1_O_3_ under different magnification. (**a**) 100 K; (**b**) 50.0 K; (**c**) 20.0 K; (**d**) 10.0 K.

Figure 2 exhibits the structural features XRD of Au:SmFe_0.9_Zn_0.1_O_3_. We can draw a conclusion that the material shows a single perovskite structure in contrast with the standard PDF card: 74-1474. However, there is no Au and Zn element characteristic peak in figure due to low doping amount. To prove the existence of above two elements, the analysis of XPS is done in Figure 3. It is observed that the Au and Zn elements are in the material. After calculation, the Au doping amount is approximately 2.6%, which is conformed to our initial doping amount. The Au 4f_7/2_ at 83.88 eV and Au 4f_5/2_ at 87.68 eV; Zn 2p_3/2_ at 1022.28 eV and 2p_1/2_ at 1045.28 eV; Sm 3d_5/2_ at 1083.38 eV and Sm 3d_3/2_ at 1109.98 eV; Fe 2p_3/2_ at 716.58 eV and 2p_1/2_ at 730.38 eV; O 1s at 529.38 eV and 531.58 eV. Figure 3b shows the Au 4f_7/2_ (83.88 eV) and Au 4f_5/2_ (87.68 eV) signals with a spin-orbit separation of −3.8 eV. In Figure 3c, two peaks can be ascribed to Zn 2p. In Figure 3d, the peaks located at about 1083.38 eV and 1109.98 eV could be assigned to 3d_5/2_ and 3d_3/2_ states of Sm^3+^. The O 1s peak in Figure 3e is asymmetric, and there is a peak at 531.58 eV and a shoulder peak at 529.38 eV. Figure 3f shows Fe 2p XPS spectra for Au:SmFe_0.9_Zn_0.1_O_3_. The peaks located at about 716.58 eV and 730.38 eV could be assigned to 2p3/2 and 2p1/2 states of Fe^3+^. Figure 4 shows the microstructure of Au:SmFe_0.9_Zn_0.1_O_3_, where the four parts of the picture show the material under different magnification. Since the easy deliquescence of Au, the material present a phenomenon of agglomeration in four part of the picture. By measuring the given scale which is at the bottom right of picture, the average particle size is about 50 nm.

**Figure 5:**
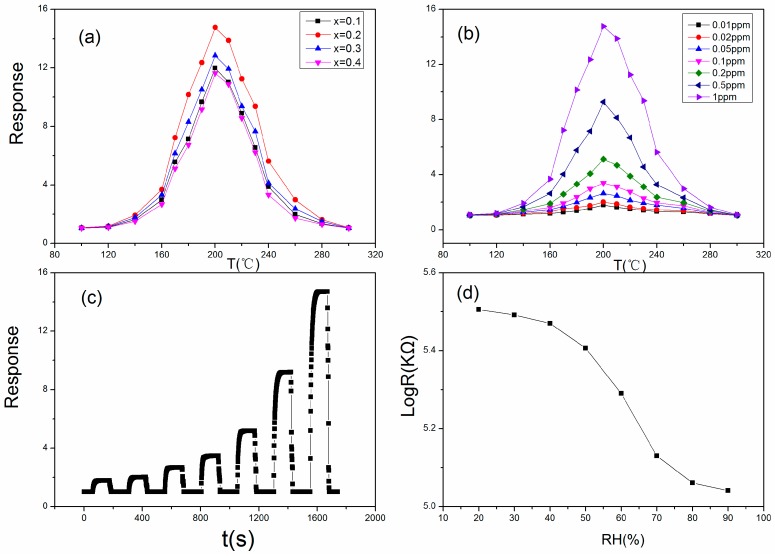
(**a**) The response of x% Au:SmFe_0.9_Zn_0.1_O_3_ (x = 0.1, 0.2, 0.3, 0.4) toward 1 ppm acetone vapor at different operating temperatures; (**b**) The operating temperature dependence of the response for Au:SmFe_0.9_Zn_0.1_O_3_ (with Ta = 750 °C) toward different concentrations of acetone gas; (**c**) The dynamic curves of response for Au:SmFe_0.9_Zn_0.1_O_3_ annealed at 750 °C toward 0.01, 0.02, 0.05, 0.1, 0.2, 0.5 and 1 ppm acetone gas at 200 °C; (**d**) The relative humidity dependence of the resistance for Au:SmFe_0.9_Zn_0.1_O_3_ (with Ta = 750 °C) at 200 °C.

Figure 5a shows the sensitivity of SmFe_0.9_Zn_0.1_O_3_ with x% Au doping (x = 0.1, 0.2, 0.3, 0.4) toward 1 ppm acetone vapor at different operating temperature. When at the very low or high temperature, the sensor show no sensitivity toward acetone vapor. The suitable temperature range of sensor is probably in 140–260 °C. The sensor is p-type due to the response is greater than 1. As the temperature of the sensor rises, the sensitivity toward acetone vapor rises. SmFe_0.9_Zn_0.1_O_3_ with 2% Au doping presents the max response, which is 14.76 at 200 °C. The response of our sensor to acetone is high in most of sensor that have been reported, and indicates that the Au:SmFe_0.9_Zn_0.1_O_3_ is a very interesting potential material in the industrial application area in the near future.

As the temperature further rises, the sensitivity of the sensor decreases. When the temperature exceeds 260 °C, the sensor shows very low sensitivity. After the temperature is above 280 °C, the sensor shows no sensitivity toward acetone vapor. There are absorbed oxygen on surface of sensor, which absorbs from outside air. Meanwhile, the absorbed oxygen also desorbs from surface of sensor to outside air. But, the rate in oxygen absorbing is larger than that in oxygen desorbing at low temperature, much absorbed oxygen molecule left. The absorbed oxygen will cause a series of chemical reactions to become oxygen species. The chemical reaction processes are listed below:

O_2_ (gas) → O_2_^−^ (ads) (1)

O_2_^−^ (ads) → O^−^ (ads) (2)

O^−^ (ads) → O_2_^−^ (ads) (3)

where (ads) means absorbed oxygen species.

After the acetone vapor is introduced, the oxygen species contain O^−^ and O_2_^−^ that will react with acetone molecules, resulting in the rising sensitivity of the sensor. With the rising temperature, the rate of oxygen absorption and desorption are improved, and the rate of the former is improved more that the latter, therefore, the amount of oxygen species rises. At 200 °C, the amount of oxygen species will reach the maximum value, which means that the chemical reaction between acetone molecules and oxygen species is the most efficient. The direct result is that the sensor shows its max sensitivity towards acetone vapor. On the other hand, the energy from the temperature provides the activation energy of the reaction, and the electron transfer in the chemical reaction will reach a max value. This is also an important factor to improve the sensitivity toward acetone vapor. As the temperature rises further, the rate of oxygen desorption improved is obviously by the energy provided by the temperature. More than that, the acetone vapor begin to desorb from the sensor surface. Little amount of acetone molecules reacts with oxygen species, resulting in a declining trend in the sensitivity.

The sensitivities toward different concentrations of acetone vapor at different operating temperatures are exhibited in Figure 5b. We measure these concentrations from low to high (0.01–1 ppm) in order. The responses are 1.78 (0.01 ppm), 2.01 (0.02 ppm), 2.63 (0.05 ppm), 3.39 (0.1 ppm), 5.13 (0.2 ppm), 9.28 (0.5 ppm) and 14.76 (1 ppm) at 200 °C. The detection limit can reach 0.01 ppm, which is extremely low. This advantage can help expand its application areas.

Figure 5c exhibits a dynamic sensitivity curve when the sensor toward different concentrations of acetone vapor. The response and recovery time is about 23 s and 7 s when the sensor responds toward 1 ppm of acetone vapor. The response and recovery time are characteristics of a sensor. It is different for one sensor toward different concentrations of tested gases or different sensors exposed to different tested gases of the same concentration. For Au:SmFe_0.9_Zn_0.1_O_3_ sensors, desorption is easier than absorption, bringing about a shorter recovery time. Exposed to 0.01 ppm, which is an ultralow concentration for acetone vapor, few sensors can achieve this detection accuracy. This indicates that the Au:SmFe_0.9_Zn_0.1_O_3_ sensor will be a very promising acetone sensor. Since the sensitivity *S* is R_g_/R_a_, a change of sensitivity also means a change of resistance of the sensor. In the primary experiments, the resistance of the sensor maintains relatively stable conditions. A large number of absorbed oxygen species absorb energy from the temperature to capture electrons from the sensor to form other oxygen species, resulting in a large number of holes with positive electricity formed on the sensor. This also indicates from other point of view that the sensor show little sensitivity toward other gases (N_2_, etc.) under a glass cover.

When the sensor accesses the tested acetone vapor, a series of chemical reactions between acetone molecules and oxygen species occur, releasing electrons which will combine with holes, resulting in a rapid decrease in the amount of holes, leading to a quick rise of the resistance. The resistance of the sensor quickly reaches a maximum, and stays stable. The chemical reactions may be summarized as follows [51]:

CH_3_COCH_3_ (vapor) + 8O^−^ → 3CO_2_ + 3H_2_O + 8e^−^ (4)

e^−^ + hole → null (5)

The resistance of the sensor decreases the relative humidity in air increases, which is presented in Figure 5d. At low relative humidity, there is little impact on the sensor and the sensitivity changes little. As the relative humidity further rises, the sensor is heavily influenced. The most intuistic conclusion is that the sensitivity decreases rapidly. When the relative humidity reaches 80%, the sensitivity decline tends to be gentle. At high temperature, H_2_O molecules will trap electrons from the sensor to react with absorbed O_2_, forming OH^-^ groups instead of H_2_O and leaving holes on the surface of the sensor. As the amount of holes increases, the resistance of the sensor decreases. Therefore, at low relative humidity, a small number of H_2_O molecules participate in reactions with absorbed O_2_, resulting in a limited number of increasing holes and the resistance of sensor changes little. At high relative humidity, the number of H_2_O molecules which will participate in reactions with absorbed O_2_ increases, leading to a rapid decrease in the resistance of the sensor.

**Figure 6:**
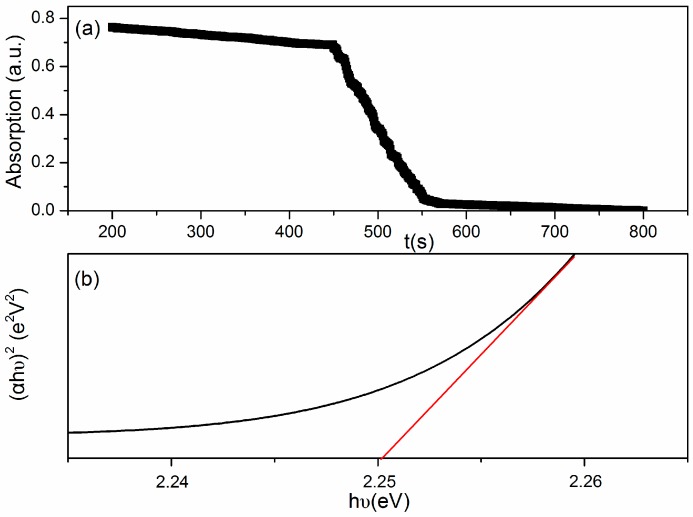
(**a**) The UV−visible diffuse reflectance spectra; (**b**) The energy band gap of the as-prepared Au:SmFe_0.9_Zn_0.1_O_3_ samples.

The high operating temperature has a serious impact on its practicality in real life. For achieve a low operating temperature, light illumination with different wavelengths was introduced in the measuring system. The optical band gap of Au:SmFe_0.9_Zn_0.1_O_3_ is shown in Figure 6. Since it is not transparent, the corresponding UV−visible diffuse reflectance spectra is recorded and presented in Figure 6a. Through the formula (αhv)^1/n^ = A(hv − E_g_), Figure 6b have been obtained. From the X axis we know that the optical band gap is approximately 2.25 eV.

**Figure 7:**
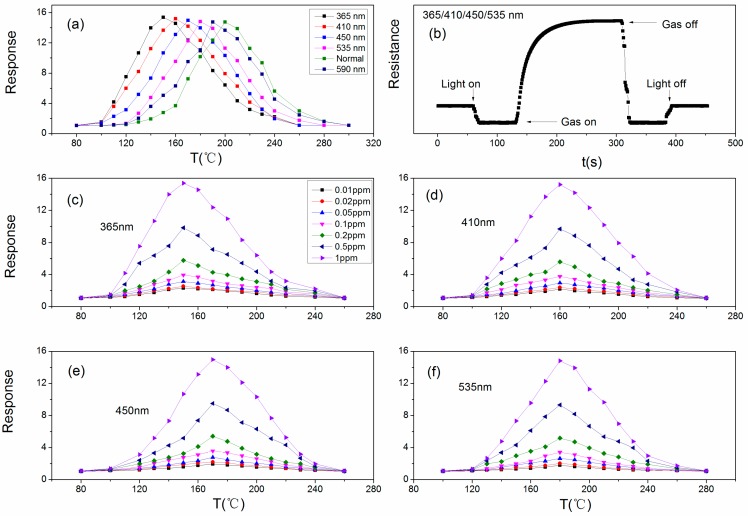
(**a**) The operating temperature dependence of response for Au:SmFe_0.9_Zn_0.1_O_3_ (with Ta = 750 °C) toward 1 ppm acetone vapor with different wavelengths of light illumination (365/410/450/535/590 nm); (**b**) The dynamic resistance curve for Au:SmFe_0.9_Zn_0.1_O_3_ exposed to acetone vapor at optimum operating temperatures when sensor under light illumination; (**c**–**f**) The temperature dependence of response for Au:SmFe_0.9_Zn_0.1_O_3_ (with Ta = 750 °C) sensor to acetone gas with different concentrations under 365/410/450/535 nm wavelengths of light illumination.

In this work, 365/410/450/535/590 nm irradiation was used test to irradiate the sensor. The results are presented in Figure 7a where the normal line is the sensor without the irradiation. The operating temperatures are reduced when under light illumination, and the operating temperature reduce more with the smaller light wavelengths. The optimum temperatures are 150 °C (365 nm), 160 °C (410 nm), 170 °C (450 nm), 180 °C (535 nm) and 190 °C (590 nm). The most important and unexpected thing is that the sensitivity of sensor is improved, and the smaller the wavelength, the greater the sensitivity of the sensor toward acetone vapor. The responses are 15.38 (365 nm), 15.2 (410 nm), 14.97 (450 nm), 14.81 (535 nm) and 14.76 (590 nm). When the sensor was irradiated by 590 nm wavelength light illumination, the sensitivity value shows no change compared with the sensor without irradiation. This may be related with the optical band gap of Au:SmFe_0.9_Zn_0.1_O_3_ and the photon energy from light illumination. The photon energy that sensor can absorb is obtained through the formula: E_g_ = 1240/λ (λ is the wavelength of light). The photons’ energies are 3.39 eV (365 nm), 3.02 eV (410 nm), 2.75 eV (450 nm), 2.31 eV (535 nm) and 2.10 eV (590 nm). Only the photon energy from 590 nm light illumination is lower than the optical band gap of Au:SmFe_0.9_Zn_0.1_O_3_, therefore, we can draw a conclusion that the sensitivity can be influenced or improved when the photon energy is larger than band gap of the sensor. Therefore, 365/410/450/535 nm light illumination are studied in the next article.

Figure 7b exhibits a dynamic sensor resistance change curve when under light illumination. The change trends of the resistance are about the same. The resistance of the sensor remains in a stable state before the light illumination is introduced. When the light illumination is introduced in the measuring system, the resistance will soon show a rapid fall, and reach a stable value. The sensor absorbs energy from the light illumination and generates electron-hole pairs. Light-induced holes from on the surface of the sensor, and capture electrons from oxygen species, which will become oxygen molecules to leave from sensor by desorption. However, the number of oxygen species on the surface of the sensor is large, especially for p-type Fe–based perovskites, so many of the oxygen species left. When acetone vapor is injected into the glass, the resistance rises quickly and reaches a stable value.

The products produced by chemical reactions between acetone molecules and oxygen species will react with acetone molecules to trap electrons. The light-induced holes can trap OH^−^ from acetone to form OH•, which has strong oxidazability. OH• can quicken the reaction between acetone molecules and oxygen species. The amount of holes decreases, and the resistance of the sensor increases. The reaction that may occur are as follows:

Au:SmFe_0.9_Zn_0.1_O_3_ + hv → e^−^ + h^+^ (6)

h^+^ + O_2_^−^ → O• (7)

h^+^ + OH^−^ → OH• (8)

CH_3_COCH_3_ (vapor) + O• + OH• → CO_2_ + H_2_O + e^−^ (9)

Where *hv* means absorbed energy from the light illumination; h^+^ means the light-induced hole.

The sensitivities toward different concentrations of acetone vapor at different operating temperatures under different wavelength light illumination are exhibited in Figure 7c–f. The responses at the optimum operating temperature are 2.31, 2.52, 3.13, 3.95, 5.77, 9.83 and 15.38 (150 °C, 365 nm); 2.17, 2.38, 2.95, 3.76, 5.59, 9.68 and 15.2 (160 °C, 410 nm); 1.95, 2.2, 2.79, 3.58, 5.42, 9.5 and 14.97 (170 °C, 450 nm); 1.79, 2.03, 2.65, 3.42, 5.18, 9.32 and 14.81 (180 °C, 535 nm). From the data, we can draw a conclusion that lower wavelength light illumination can obtain better sensor sensitivity. The optimal light wavelength is 365 nm.

**Figure 8:**
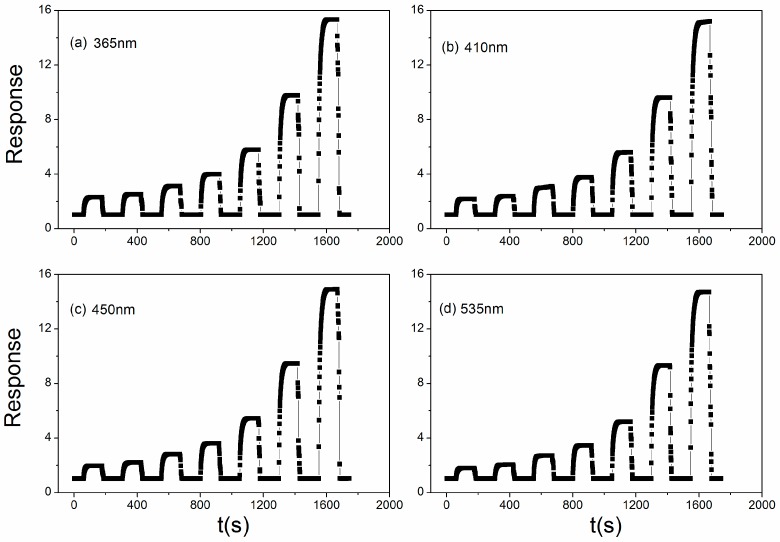
The dynamic sensitivity curves for Au:SmFe_0.9_Zn_0.1_O_3_ to 0.01, 0.02, 0.05, 0.1, 0.2, 0.5, and 1 ppm acetone irradiated by light illumination: (**a**) 365 nm; (**b**) 410 nm; (**c**) 450 nm; (**d**) 535 nm.

Figure 8 shows the dynamic response of the sensor to 0.01, 0.02, 0.05, 0.1, 0.2, 0.5, and 1 ppm acetone under different light illumination wavelengths. Figure 8a: 365 nm; b: 410 nm; c: 450 nm; d: 535 nm. The sensor shows the short response and recovery time when under light illumination, which can add to its ability in industry applications in the future. From Figure 8a, we can see that the response and recovery time is about 20 s and 6 s when the sensor exposed to 1 ppm acetone, and the times above are lower than the initial value when without light illumination, but the response and recovery time of the sensor toward acetone vapor changes little when influenced by light illumination.

**Figure 9:**
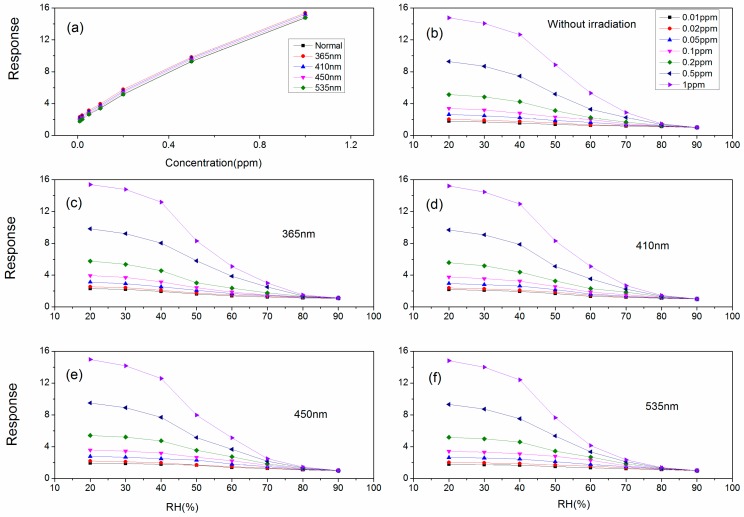
(**a**) The response dependence on the acetone vapor concentration when sensor under different wavelengths light illumination; (**b**–**f**) The humidity dependence of the sensing response for Au:SmFe_0.9_Zn_0.1_O_3_ annealed at 750 °C toward different concentrations of acetone vapor.

As the concentration (0.1–1 ppm) of acetone vapor increases, the sensitivity of the sensor increases, which is shown in Figure 9a where the normal trace is the sensor without the irradiation. Whether under light illumination or not, the dynamic trends are the same. As the relative humidity increases, the sensitivity of the sensor which is under light illumination decreases, which is shown in Figure 9b–f. With the same change trend as the sensor resistance, the sensitivity changes little at low relative humidity. As the relative humidity increases, the sensitivity rapidly decreases. When the relative humidity reaches 90%, the sensor shows no sensitivity toward acetone vapor.

From the above we know that the chemical reaction products between acetone molecules and oxygen species is H_2_O and CO_2_. Therefore, this chemical reaction will be weakened with the increasing relative humidity.

**Figure 10:**
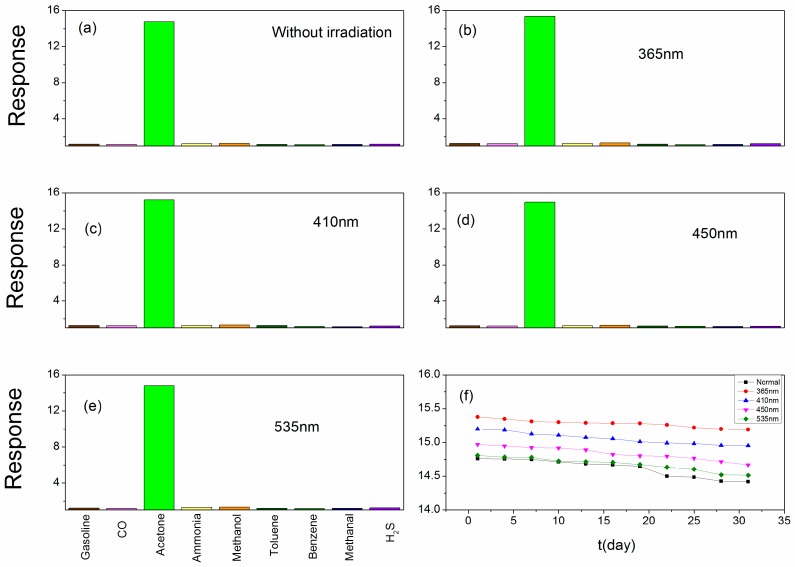
(**a**–**e**) The response of sensor based on Au:SmFe_0.9_Zn_0.1_O_3_ toward 1 ppm different tested gases under light illumination (365/410/450/535 nm) compared with the sensor without irradiation at different optimum operating temperatures. (**f**) The response stability of sensor based on Au:SmFe_0.9_Zn_0.1_O_3_ annealed at 750 °C under different wavelengths of light illumination at different optimum operating temperatures toward 1 ppm acetone vapor for a month.

In a practical application process, many different kinds of gases may be together. For accurate detection of acetone content in a mixture of gases, the sensor should not be impacted by other gases. Therefore, checking out the selectivity of the sensor is important for its use in real life. Figure 10a–e show the selectivity of the sensor toward 1 ppm of different gases under light illumination. It can be observed from the picture that the sensor shows greatly selectivity toward acetone compared with other gases. The acetone molecule has a larger dipole moment (2.88 D) [52,53], which makes it more easily adsorbed on our nano-Au:SmFe_0.9_Zn_0.1_O_3_ with its electric dipole moment. In addition, the functional group of acetone is a carbonyl and the C-C and C-H groups in acetone can be easily broken. Both factors may result in a high selectivity of the sensor toward acetone vapor. Stability is also important in practical applications. A good stability sensor will have a larger application scope. To measure the stability of the proposed sensor, the following measurement is done. The sensor is measured every three days for a month, and stored in a vacuum bag after every measurement. Figure 10f shows the experimental results of the sensor stability tests. It is observed that the sensor has good stability whether irradiated or not. However, the sensor shows better stability when under light illumination, and the smaller the wavelength of the light, the better the stability is.

## 4. Conclusions

Au:SmFe_0.9_Zn_0.1_O_3_ is synthesized by a sol-gel method and annealed at 750 °C. The sensitivity of this sensor is quite high among materials that have been reported. As the relative humidity increases, the resistance and sensitivity of the sensor declined. For reducing the optimum operating temperature, light illumination with different wavelengths was introduced. The sensitivity is improved and the optimum operating temperature is decreased. In this work, the optimal light wavelength for the highest sensitivity and lowest optimum operating temperature of the sensor is 365 nm. Through dynamic resistance curves, when the sensor irradiated by light, an improved sensitivity mechanism has been observed. More than that, the sensor has good selectivity toward acetone with an absolute advantage compared with other gases. The sensor shows better sensitivity and stability when under light illumination.
